# Discovery of Novel Druggable Sites on Zika Virus NS3 Helicase Using X-ray Crystallography-Based Fragment Screening

**DOI:** 10.3390/ijms19113664

**Published:** 2018-11-20

**Authors:** Ali Munawar, Steven Beelen, Ahmad Munawar, Eveline Lescrinier, Sergei V. Strelkov

**Affiliations:** 1 Biocrystallography, Department of Pharmaceutical and Pharmacological Sciences, KU Leuven, 3000 Leuven, Belgium; ali.munawar@kuleuven.be (A.M.); steven.beelen@kuleuven.be (S.B.); 2 Orthogon Therapeutics LLC, Canton, MA 02021, USA; amunawar@orthogontherapeutics.com or ahmad@orthogontherapeutics.com; 3 Medicinal Chemistry, Department of Pharmaceutical and Pharmacological Sciences, KU Leuven, 3000 Leuven, Belgium; eveline.lescrinier@kuleuven.be

**Keywords:** Zika virus, flaviviruses, NS3 helicase, antivirals, structure-based drug design, X-ray crystallography, fragment screening, drug discovery, protein–protein interaction inhibitor, allosteric inhibitor

## Abstract

The flavivirus family contains several important human pathogens, such as Zika virus (ZIKV), dengue, West Nile, and Yellow Fever viruses, that collectively lead to a large, global disease burden. Currently, there are no approved medicines that can target these viruses. The sudden outbreak of ZIKV infections in 2015–2016 posed a serious threat to global public health. While the epidemic has receded, persistent reservoirs of ZIKV infection can cause reemergence. Here, we have used X-ray crystallography-based screening to discover two novel sites on ZIKV NS3 helicase that can bind drug-like fragments. Both sites are structurally conserved in other flaviviruses, and mechanistically significant. The binding poses of four fragments, two for each of the binding sites, were characterized at atomic precision. Site A is a surface pocket on the NS3 helicase that is vital to its interaction with NS5 polymerase and formation of the flaviviral replication complex. Site B corresponds to a flexible, yet highly conserved, allosteric site at the intersection of the three NS3 helicase domains. Saturation transfer difference nuclear magnetic resonance (NMR) experiments were additionally used to evaluate the binding strength of the fragments, revealing dissociation constants (K_D_) in the lower mM range. We conclude that the NS3 helicase of flaviviruses is a viable drug target. The data obtained open opportunities towards structure-based design of first-in-class anti-ZIKV compounds, as well as pan-flaviviral therapeutics.

## 1. Introduction

The recent outbreak of Zika virus (ZIKV), a mosquito-borne flavivirus, in the Americas and the Pacific, triggered a public health crisis, leading the World Health Organization to declare ZIKV infections as a global emergency [1]. Over 1.67 M cases were reported in Brazil [2], with another 41,957 cases in the United States and its territories [3]. This wave of ZIKV infections resulted in a sudden rise in neurological complications, the most severe of which are microcephaly or congenital fetal malformation in children born to infected women [4], and Guillain–Barre syndrome [5]. There are, at present, no approved antivirals or vaccines to address this medical problem. Effective, direct-acting, and safe anti-ZIKV therapeutics are required to reduce the viral load, limit disease severity, and prevent or minimize associated complications. Moreover, when administered prophylactically, such antivirals may suppress future outbreaks.

**Figure 1:**
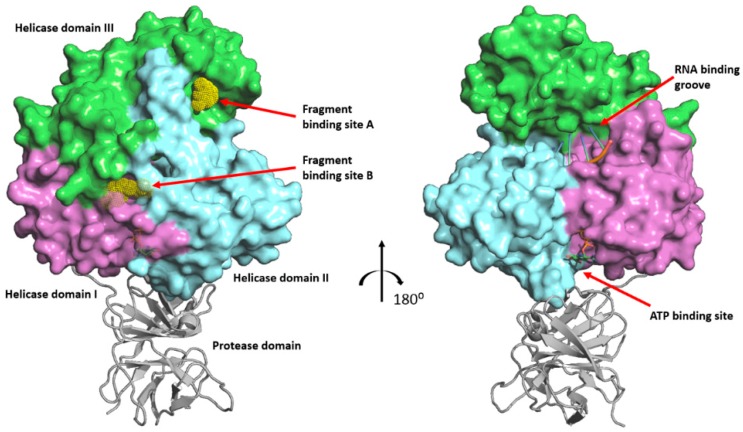
Overall structure (two views related by 180° rotation, black arrow) of ZIKV NS3 protein, including the helicase domains I (magenta surface), II (cyan surface), and III (green surface), as well as the protease domain (grey ribbons). The latter is approximated from the homologous DENV NS3 full-length structure (PDB code 2VBC), which has been superposed on the ZIKV NS3-Hel structure. The binding positions of RNA and ATP are also shown. The fragment-binding sites, A and B, discovered here, are indicated using the ball representation for fragments ***1*** and ***3*,** respectively.

Just like the hepatitis C virus, ZIKV is an RNA virus belonging to the Flaviviridae family [6]. Within this family, several viruses of the *Flavivirus* genus, including ZIKV, dengue (DENV), West Nile (WNV), yellow fever (YFV), Japanese encephalitis (JEV), Murray Valley encephalitis (MVEV), and tick-borne encephalitis (TBEV) viruses, pose a serious threat to human health [7]. The genomes of all these viruses encode a polyprotein that is processed into three structural proteins and seven nonstructural (NS) proteins (NS1, NS2A, NS2B, NS3, NS4A, NS4B, and NS5) by both host proteases and the viral NS2B-NS3 protease [8]. The NS3 protein consists of an N-terminal protease domain (NS3-Prot) followed by a helicase (NS3-Hel), both of which are vital for viral replication. The helicase is formed by three tightly associated domains of approximately equal size [9] (Figure 1). NS3-Hel belongs to the helicase superfamily SF2 [10], possessing RNA-stimulated ATPase activity, which results in the unwinding of viral RNA. The NS3-Hel forms a replication complex (RC) with NS5 polymerase. This complex is responsible for the replication of viral genome [9]. Inactivation of RC in both DENV and hepatitis C virus renders them incapable of proliferation [11,12,13].

Crystal structures of ZIKV NS3-Hel in the apo, ATP, and single-stranded RNA-bound (ssRNA) forms have shed light on the enzyme’s function at a molecular level [14]. Overall, the three-domain structure of NS3-Hel is highly conserved across all flaviviruses. For instance, ZIKV domains I, II, and III superimpose on the equivalent domains of DENV4 with root mean square deviations in atomic positions of 0.60, 0.31, and 0.40 Å, respectively [14]. Domain I of ZIKV NS3-Hel includes a flexible P-loop or Walker A motif, which is critical for NTP binding and catalysis to power helicase activity [9]. The ssRNA predominantly interacts with domains II and I, binding to a groove separating these two domains from domain III [9]. Upon ssRNA binding, the NS3-Hel undergoes significant conformational changes, whereby domain II and III rotate and move 9 Å away from domain I [15]. This rotation enables the helicase to act as a “double-leaf swing gate”, with each leaf opening in a reverse direction to the other, enlarging the groove to accommodate the ssRNA [9].

In general, helicases are considered an attractive antiviral target, due to their central role in viral replication [9,16,17]. While several antivirals are known to act on the NS3-protease domain [18,19,20], the discovery of targeted NS3-Hel inhibitors has been elusive [21]. Experience from two decades of NS3-Hel discovery campaigns in hepatitis C virus have revealed the main hurdles along this way. In particular, high-throughput screening (HTS) assays, monitoring helicase-catalyzed RNA unwinding, suffer from low hit rates and false positives [15,22]. In the past, the ATP- and nucleotide-binding cavities were suggested to be the only pockets suitable for drug binding [15].

Accordingly, we set out to perform fragment-based screening (FBS) to identify novel, medicinal starting points for the design of ZIKV NS3-Hel inhibitors. FBS has emerged as a powerful alternative to HTS for rapid discovery and subsequent elaboration of hits into quality lead compounds [23]. However, a major challenge with conventional FBS has been the lack of sensitive screening techniques to detect weakly bound hits. For instance, Coutard et al. concluded that the highly homologous DENV NS3-Hel may not be amenable to FBS [24]. The authors obtained 36 fragment hits through dynamic scanning fluorimetry, however, none of these hits could be validated using X-ray crystallography.

Here, we demonstrate the potential of direct fragment-based screening by X-ray crystallography (FBS-X) applied to ZIKV NS3-Hel. This approach has recently gained considerable attention [25,26,27]. Although laborious, FBS-X is not only highly reliable and sensitive as an FBS technique, but also provides two additional benefits, namely (a) identification of the precise fragment-binding site on the target protein, and (b) deliverance of atomic resolution information on the fragment-binding pose and interactions. Therefore, FBS-X data can be directly exploited towards rational drug design.

Our FBS-X-based efforts with ZIKV NS3-Hel (corresponding to residues 171–617 of the full-length NS3 protein) have resulted in the discovery of two novel, mechanistically relevant binding sites, each accommodating two fragment hits. To our knowledge, this is the first report that identifies druggable binding sites for NS3-Hel of ZIKV or related flaviviruses. These results provide detailed structural information towards subsequent medicinal chemistry optimization.

## 2. Results

### 2.1. FBS-X Reveals Two Novel Drug Binding Sites on ZIKV NS3-Hel

To conduct an FBS-X study, it was imperative to develop an efficient protocol towards *Escherichia coli* overexpression and purification of ZIKV NS3-Hel (see Materials and Methods for details). The obtained mg quantities of highly pure enzyme have been used to screen for crystallization conditions. After several rounds of optimization, single 3D crystals that diffracted X-rays to beyond 2.5 Å resolution could be reproducibly obtained, which was a prerequisite for subsequent FBS-X experiments.

**Figure 2:**
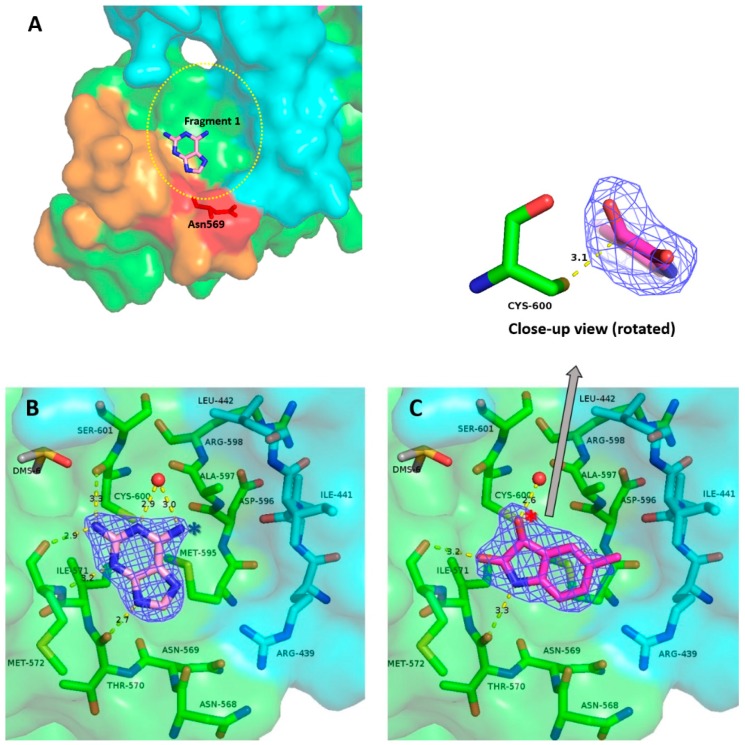
Fragment-binding site A. (**A**) Overall view of the binding pocket shown as a solvent-accessible surface with fragment ***1*** in place. The rim of the pocket is approximated by an oval (yellow dotted line). The surface of residues 565 to 584 is colored orange. Residues Asn568, Asn569, and Thr570 are highlighted in red. The rest of domain III is in green. Domain II is colored cyan. (**B**) View of the binding pocket. The majority of the residues forming the pocket are from domain III (green) and some are from domain II (cyan). Binding pose of fragment ***1*** is shown as sticks. Polder electron density map for the fragment is drawn as blue mesh at 4σ. H-bonds made by the fragment are shown by dashed lines. Ordered water molecules interacting with the fragment are shown as red spheres. Blue asterisk indicates an amino group in C6 position, discussed in the text. (**C**) Corresponding electron density and H-bonds made by fragment ***2*.** On top of the figure, a rotated close-up view of fragment ***2*** is shown to illustrate the off-plane position of the oxygen at C3 (marked with a red asterisk). The distance between the sulfur atom of the Cys600 residue and the C3 atom of the fragment is shown by a dashed line.

**Figure 3:**
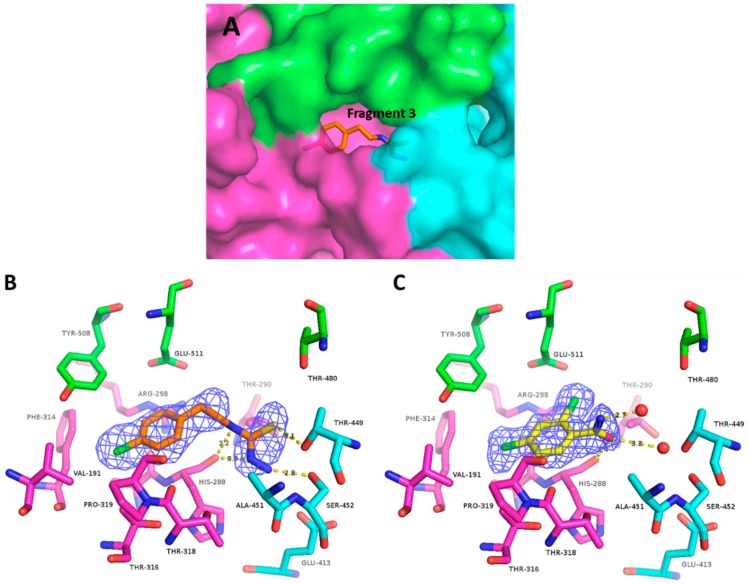
Fragment-binding site B. (**A**) Overall view of the binding pocket with fragment ***3*** in place. Coloring for the three helicase domains is the same as in Figure 1. (**B**) Electron density map and H-bonds made by fragment ***3***. Residue Pro320 locates in this view on top of the fragment, and has been omitted for clarity. (**C**) Electron density and H-bonds made by fragment ***4***. Residue Pro320 has been omitted for clarity.

Next, a total of 200 drug-like fragments were randomly chosen from two diverse commercial libraries. These fragments were used to soak the obtained crystals without visible deterioration of the latter in most cases. Statistics of the FBS-X experiments is given in Table 1. Diffraction datasets for 88 fragment soaks were obtained at better than 3 Å resolution. Subsequent structure solution was based on the previously established crystal structure of ZIKV NS3-Hel [14]. As a result, six instances of drug-like fragments bound to ZIKV NS3-Hel were established. Two of these fragments were detected in-between symmetry-related protein molecules. Being a likely consequence of binding between a specific crystal contact, these two fragments were eliminated from further analysis, in line with standard practice [28]. The remaining four fragment binders (discussed below) were confirmed by repeating the crystal soaking and X-ray experiments (Table 2). The obtained electron density maps unambiguously resolve the binding pose of all four fragments (Figure 2B,C and Figure 3B,C).

**Figure A1:**
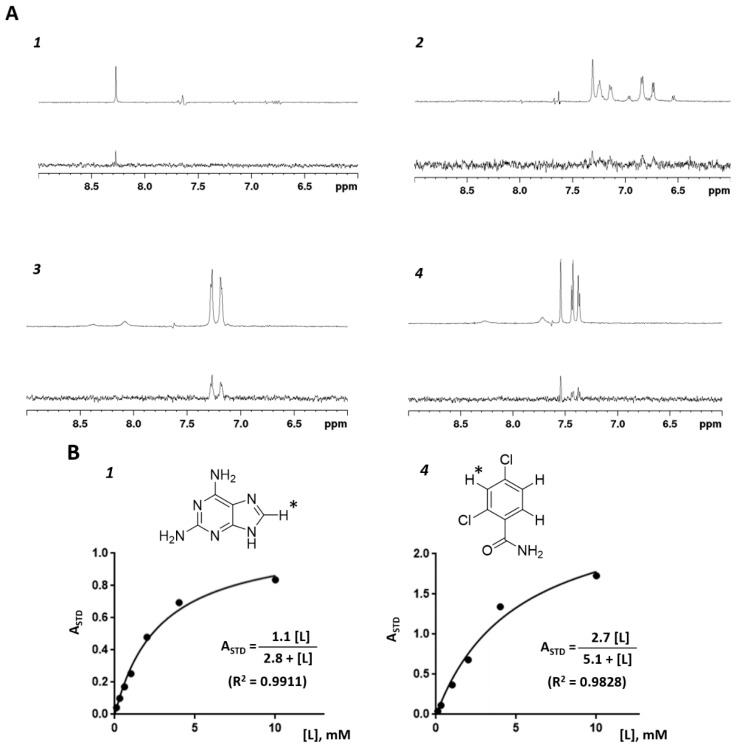
Saturation transfer difference (STD) studies. (**A**) Spectra illustrating the binding of four fragments at 1 mM concentration to ZIKV NS3-Hel. For each fragment, reference ^1^H NMR spectrum is shown on top, and the STD spectrum is shown at the bottom. (**B**) STD amplification factors A_STD_ plotted vs the ligand concentration for fragments ***1*** and ***4***. The plots are based on singlet peaks which arise from the protons, indicated with a star on the structural diagrams (top).

Binding of the four fragments identified through FBS-X was further studied in solution using 1D saturation transfer difference (STD) NMR experiments. At 1 mM concentration, all four fragments produced a distinct binding signal with 50 µM helicase (Figure A1, panel A). In addition, for fragments ***1*** and ***4*** STD experiments were repeated upon titrating them in the 0.1 mM to 10 mM range, yielding the dissociation constants K_D_ of 2.8 ± 0.4 mM and 5.1 ± 1.4 mM, respectively (Figure A1, panel B).

These four fragment hits were found to bind at two discrete sites on the NS3-Hel (Figure 1). Neither of the two sites have previously been reported as accommodating any ligands, and application of drug pocket-searching computational tools did not detect these sites [14]. Interestingly, both sites are away from the RNA-binding cleft and the ATP-binding region. Fragments ***1*** and ***2*** both bind in a surface pocket on helicase domain III, which we refer to as binding site A. Fragments ***3*** and ***4*** bind in a tunnel-shaped cavity located at the interface of all three domains, which are referred to as site B.

### 2.2. Site A Is at a Critical Interface of the NS3-NS5 Flaviviral Replication Complex (RC)

Site A is a relatively shallow cavity with a solvent accessible surface area (*SASA*) of 522 Ȧ^2^ as computed using PyMOL (Schrodinger LLC, New York, USA) (Figure 2A). Most of the residues forming the cavity belong to the C-terminal portion of NS3-Hel domain III. These residues are Asn568 through Met572, Met595, Ala597, Cys600, and Ser601 (Figure 2B,C). In addition, residues Arg439, Val440, and Ile441, coming from domain II, form part of the “rim” around the cavity. The “floor” of the pocket is formed by the hydrophobic side chains of Ile571, Met595, Ala597, and Cys600, whereas the rim is assembled from mostly polar side chains.

Binding site A is located at the interface between NS3 and NS5 proteins, which are known to interact upon the formation of a replication complex (RC). The RC is an essential feature of all flaviviruses, as it simultaneously unwinds and extends the viral RNA [29,30]. Indeed, studies in DENV have previously indicated that the NS3-Hel interacts with the “thumb” region of the RNA-dependent RNA polymerase (RdRp) domain of the NS5-polymerase [31]. The C-terminal 50 residues of NS3-Hel (which are responsible for the site A formation) are important for interacting with NS5 and, consequently, viral replication [31]. This same study showed that a DENV NS3 peptide, spanning residues 566–585 (corresponding to residues 565–584 in ZIKV NS3, Figure 2A) competitively disrupts the NS3–NS5 interaction, and that a single Asn570Ala substitution abolishes the NS3–NS5 interaction, terminating viral RNA synthesis and infection [31]. In ZIKV NS3-Hel, the corresponding residue is conserved (Asn569), and contributes to the rim surrounding the site A (Figure 2A). Hence, the data obtained for DENV indicate that site A is highly relevant for the normal functioning of NS3-Hel.

In agreement with its role in the RC formation, binding site A reveals multiple features that are typical for protein–protein interaction (PPI) “hotspots”. Indeed, the predominantly apolar floor of the cavity is reminiscent of PPI interfaces that tend to possess patches of hydrophobic surfaces of 200–400 Å^2^. In addition, the more polar rim of this pocket includes the residue Arg439, which is conserved in most flaviviral helicases (Table A1). Arginines and lysines are frequently observed interfacial residues involved in PPIs [32], as such residues can guide intermolecular recognition leading to a large decrease in *SASA* upon complex formation [33]. Several additional polar and non-polar residues, i.e., Asp569, Ile571, Ile441, and Ala597, forming the rim, are also well conserved across various flaviviruses (Table A1).

### 2.3. Binding of Fragments **1** and **2** to Site A Offers Multiple Drug Design Prospects

#### 2.3.1. Fragment ***1***

Fragment ***1***(Figure 2B) is 2,6-di-aminopurine. The double ring structure of the fragment enables a π-alkyl interaction with the floor of the pocket. This purine ring also enables several hydrogen bonds with residues in the pocket. Specifically, the 2-amine of ***1*** acts as a hydrogen bond donor to the carbonyl groups of Met572 and Cys600. The secondary amine in position 7 of the purine also forms a hydrogen bond with the main-chain carbonyl of Thr570. A water molecule is held in place by hydrogen bonding with the 6-amine and 5-nitrogen of the purine. Atom C8 is involved in a carbon hydrogen bond with the carbonyl of Asn568. There is also an ordered DMSO molecule in the vicinity of the 2-amine group of ***1***, providing a possible fragment-linking opportunity.

Vincetti and co-workers recently described the anti-DENV effect of di-aminopurine derivatives that were identified through in silico docking of compounds against the DENV NS5 polymerase [34]. While no structural evidence of binding to NS5 was shown, the authors hypothesized that the compounds may disrupt the NS3–NS5 RC in DENV by binding to NS5. Of note, their best-performing antiviral compound, ***16i***, had an aminophenol substitution at the C6 position, compared to just an amino group in our fragment ***1*** (indicated in Figure 2B with a blue asterisk). Compound ***16i*** can be accommodated in this binding pocket without any obvious steric clashes. It is conceivable that compound ***16i*** may disrupt the RC complex through binding of NS3-Hel instead of NS5. We thus conclude that fragment ***1*** presents a promising scaffold that can be grown to produce more potent lead molecules in the future.

#### 2.3.2. Fragment ***2***

Fragment ***2*** (Figure 2C) is 5-methyl-2,3-dihydro-1H-indole-2,3-dione, also known as 5-methylisatin. Like ***1***, this fragment enables *π*-alkyl stacking against the pocket floor. The toluene group of ***2*** appears to favor the hydrophobic side of the pocket reaching out to Ile441. A notable feature of this binding mode is the proximity of the C3 atom of ***2*** to the nucleophilic thiol group of Cys600. Moreover, the electron density map reveals that the oxygen atom linked to C3 (this atom is indicated with a red asterisk in Figure 2C) locates off the ring plane, as evident from the close-up view provided on top of Figure 2C. Isatins are known to be capable of forming covalent adducts with cysteines [35]. In our case, however, no continuous electron density is seen between the reactive sulfur of Cys600 and the electrophilic carbon of fragment ***2*** (Figure 2D). The distance between these two atoms is 3.1Å, which is longer than is expected for a carbon–sulfur covalent bond (1.8 Å) [36]. It is plausible that either the covalent bond to Cys600 was only formed in a fraction of cases, or such a bond has been degraded during exposure to X-rays [37]. It should also be noted that our STD NMR experiments reveal several additional proton signals compared to those expected for the baseline structure of ***2***, suggesting the presence of additional chemical species. Indeed, isatin can be hydrated at the C3 position, whereby the carbonyl is converted to two OH groups [38,39]. Sulfhydryl groups of cysteine residues are generally known to engage in a range of interactions in native proteins [40]. With respect to small molecule ligands, covalent binding to cysteines is often a desired design feature, as this leads to prolonged target engagement and hence a sustained drug action [41,42].

### 2.4. Site B Is at a Flexible, Interdomain Hinge That is Highly Conserved across Flaviviruses

Site B is a relatively narrow, tunnel-like pocket which is located close to the center of the ZIKV NS3-Hel structure at the interface of the three leaf-like domains (Figure 3A). This site is on the face opposite to the RNA binding groove, and at least 13 Å away from the P-loop involved in ATP binding. Comparison of the *apo*, ssRNA, and nucleotide-bound X-ray structures has previously suggested that this hinge region grants the necessary conformational flexibility for helicase processivity [9,43].

This binding site is lined with mostly hydrophobic residues on one end (Thr316, Val191, Ala287, Phe314, Tyr508, Trp487) and polar residues (His288, Glu413, Ser452, Thr449, Gln455) on the other end (Figure 3B,C). The hydrophobic end of this pocket is comprised largely of residues from domains I and III. One side is formed by a well-ordered salt bridge between residues Arg298 (domain I) and Glu511 (domain II). The polar end of this tunnel is made up of residues from domain I and II. This end features a cavity with a considerable volume which, as reported for the HCV NS3-Hel, appears to expand or contract upon helicase activity [43]. Given the dynamic character of this site, a small molecule may be able to stabilize a particular conformation of the helicase, impairing its function. Indeed, the residues forming the tunnel-like site A are remarkably conserved across other human flaviviruses (Table A1), suggesting a critical structural and functional role [44].

### 2.5. Fragments 3 and 4 Collectively Reveal Features Enabling Efficient Binding at Site B

#### 2.5.1. Fragment ***3***

Fragment ***3*** is 3-amino-1-[2-(4-chlorophenyl)ethyl]thiourea. This fragment is well-accommodated in the pocket, with 10 out of its 13 non-hydrogen atoms making diverse interactions with all three domains of NS3-Hel (Figure 3B). The aryl chloride group of this fragment is wedged between the Pro320 residue located closer to the outside, and the main chain atoms of His288 located at the bottom of the pocket. The former interaction corresponds to the aromatic–proline stacking [45]. In the lateral direction, this group points to the hydrophobic end (Phe314, Val191, Ala287, and Tyr508) of the binding tunnel, leaving little room for growing this fragment. The aromatic ring of ***3*** is connected by an ethyl-linker to a tail comprising a thiourea group. This tail inserts itself into the polar, solvent-rich region of the tunnel, forming an interlaced H-bond network with various residues. One nitrogen of the thiourea group creates a hydrogen bond with His288, whereas the sulfur serves as a hydrogen bond acceptor to both Thr449 and Ser452. The protonated amine group at the distal end of the molecule is secured by a favorable ion pairing with the side chain of Glu413. While ***3*** appears to snugly fit the hydrophobic side of the tunnel, the polar end of the tunnel offers additional volume to accommodate multiple functional groups.

#### 2.5.2. Fragment ***4***

Fragment ***4*** is 2,4-dicholorobenzamide. The di-chlorobenzene group of this fragment has a similar binding mode to the aryl-chloride of the longer ***3*** molecule (Figure 3C). In ***4***, the para chlorine also buries itself into the hydrophobic crevice formed by Val191, Phe314, Tyr508, Ala287, whereas the additional chlorine present at the ortho position nests against Trp487 and Thr290. The amide group coordinates multiple water molecules. The OH group of the amide donates a hydrogen bond to the carbonyl group of His288. While this fragment lacks the thiourea tail of ***3***, it demonstrates that the binding pocket can accommodate a second halogen at the para position. Combining the functional groups present in ***3*** and ***4*** into a single drug molecule may be a rapid strategy to improve affinity.

## 3. Discussion

The essentiality of NS3-Hel to the flaviviral life cycle has been clearly demonstrated, making it an attractive drug target. However, despite the availability of high resolution NS3-Hel structures for several flaviviruses, there is still little knowledge of suitable sites for rational drug design. Here, we have used FBS-X to reveal two distant, previously unrecognized drug-binding sites on ZIKV NS3-Hel, which are conserved in all flaviviruses. Detailed analysis of the four drug-like fragments bound to NS3-Hel provides chemical starting points and corresponding pharmacophores for the rational development of two separate classes of helicase-targeting inhibitors. Using STD NMR experiments, we have additionally shown that compounds ***1*** and ***4***, binding to sites A and B, respectively, have affinities (K_D_) of several mM. While modest binding strengths are not unexpected for small drug-like fragments, these results further substantiate the use of both sites towards future drug design.

Specifically, our discovery of a “druggable pocket” (site A) at the interface of the NS3–NS5 heterocomplex provides an opportunity to develop inhibitors capable of disrupting the flaviviral RC, which is an attractive antiviral strategy [17]. Indeed, site A reveals features characteristic of a PPI “hotspot”. While challenging, considerable progress has been made towards the design principles of small-molecule PPI disruptors [46]. Both fragments found to bind at site A occupy only about one-third of the pocket volume, implying that each fragment can be “grown” further in several directions. Finally, the presence of a reactive Cys600 in this pocket is unique to ZIKV NS3-Hel, and provides an opportunity for the development of specific, high-affinity small molecules capable of forming a covalent link with the pocket.

Site B corresponds to a tunnel-shaped pocket at the interface of all three NS3-Hel domains. Since the helicase function is known to involve considerable interdomain motions, we hypothesize that agents targeting this pocket may compromise helicase motor processivity. The conserved nature of this pocket is a further attractive feature as it opens the possibility of pan-flavivirus inhibitors with a high genetic barrier to drug-resistance.

Future structure–function studies, focusing on mutational analysis of residues forming the two binding sites, will deepen the biological understanding of their role in ZIKV replication and helicase function. The fragment hits uncovered here represent tractable starting points for structure-guided medicinal chemistry optimization. Activity testing *in vitro* and in cell-based assays will be possible once high-affinity derivatives of these fragments are generated.

## 4. Materials and Methods

### 4.1. Fragment Libraries

The screened compounds originated from two commercially available libraries, including Zenobia Library 1 (352 compounds) and Maybridge RO3 Core Library (1000 compounds). Two hundred compounds were selected by picking approximately every 7th compound from the total list of 1352 compounds. The mean molecular weight of the compounds was 211.7 Da. The compounds possessed a combination of rigid, low-complexity structures, as well as ≥3 rotatable bonds. Both libraries were dissolved in 100% DMSO at 100 mM.

### 4.2. Protein Purification and Crystallization

ZIKV NS3-Hel (residues 171–617 of the NS3 protein) was overexpressed in a pET30+ plasmid with a cleavable, N-terminal 6× His-tagged SUMO tag using a Rosetta 2(DE3) pLysS *E. coli* strain (EMD Biosciences, San Diego, CA, USA) and Studier’s ZYP-5052 auto-induction medium [47]. The cells were grown at 37 °C until the OD_600nm_ reached 0.5, thereafter, the temperature was reduced to 16 °C, and cell growth was continued for another 24 h. Cells were lysed by sonication. The target protein was captured using nickel affinity chromatography using Nuvia^®^ beads (Bio-Rad, Hercules, CA, USA) with 50 mM sodium phosphate buffer (pH 8.0), 500 mM NaCl, and eluted with 300 mM imidazole. The tag was cleaved off with SUMO protease [48] during overnight dialysis into 20 mM Tris buffer (pH 7.0), 10% glycerol, and 5 mM DTT. The sample was further purified using anion exchange chromatography (GE HiTrap™ Q HP 5 mL column) by using a linear gradient elution with 20 mM Tris buffer (pH 7.0), 0–1 M NaCl, and 5 mM DTT. Peak fractions were pooled, concentrated, and then applied to a GE HiLoad™ 16/600 Superdex 200 pg column equilibrated with 20 mM Tris pH 7.0, 150 mM NaCl, and 5 mM DTT. The purified protein was concentrated to 8 mg/mL, and crystallized by the hanging drop technique. Hanging drops were set up using a 1.5:1 ratio of protein, and the precipitation solution, which contained 5–8% (*w*/*v*) PEG3350, 100 mM HEPES buffer (pH 7.0), and 100 mM sodium acetate. Crystals grew over 3–5 days at 18 °C. While the initial crystals appeared as thin plates, successive rounds of seeding produced more 3D crystals that diffracted at better than 2.5 Å resolution. 

### 4.3. Fragment Soaking and X-ray Data Collection

Preliminary tests indicated that soaking of the obtained ZIKV NS3-Hel crystals tolerated up to 50% DMSO without adversely impacting X-ray diffraction quality. Crystals were soaked in the crystallization mother liquor supplemented with 33 mM of the drug-like fragment and 33% DMSO (Sigma-Aldrich, Milwaukee, WI, USA), for 45 min. In cases where crystals appeared to deteriorate, soaking times were shortened to 15 min. X-ray diffraction data were collected at the F1 beamline of Cornell High Energy Synchrotron Source at 100 K using 0.95667 Å radiation and a Dectris Pilatus 6 M detector. Data were processed using *iMosflm* [49] and *XDS* [50]. Structures were solved by molecular replacement with *MolRep* [51] using PDB code 5MFX. Model building was carried out using *Coot* and refinement using *Refmac* [52], and *BUSTER* [53]. Cross-validation was done by R-free calculated using 2.5% of the reflections. Fragment hits were placed into the electron density maps using *RhoFit*. Confirmation of the correct placement of each fragment was achieved through the generation of a polder electron density map [54].

### 4.4. NMR STD Experiments

Initial measurements were done with 2.5 mg/mL (50 µM) protein and 1mM drug-like fragment in 50 mM phosphate buffer (pH 8), 100 mM NaCl, 5 mM DTT, and 1% DMSO (2% DMSO for fragment ***3***), at 5 °C. Bruker Avance II 600 NMR spectrometer equipped with a cryogenic TCI probe and a z-gradient was employed. Programs *zgesgp* [55,56] and *stddiffesgp* [56] were used for data collection. Excitation sculpting to suppress the water signal and 5 s STD saturation time was applied. In 1D proton and STD experiments, 4 and 32 scans were accumulated, respectively, for each experiment. A delay of 5.5 s was applied between each free induction decay (FID) to ensure complete relaxation. The spectra for both on-resonance and off-resonance saturation at 0.7 and 12 ppm, respectively, were collected, interleaved for all samples. The Bruker command *stdsplit* was used to process and subtract on- and off-resonance FIDs. The STD spectrum of the corresponding control sample, containing the protein solution only, was subtracted from the measured spectra to minimize the protein background.

Towards the dissociation constant (K_D_) estimation, STD measurements were repeated with 64 scans upon the titration of fragments ***1*** and ***4*** (0.1–10 mM) into 50 µM NS3-Hel solution. In each case, specific singlet peaks were analyzed (Figure A1, panel B). The resulting STD amplification factors were obtained with Topspin 3.2 software (Bruker, Billerica, MA, USA), and fitted to the equation

A_STD_ = A_STD_(∞) × [L]/(K_D_ + [L]),

where [L] is the fragment concentration.

## Figures and Tables

**Table 1:** Statistics of the FBS-X screening experiment.

Parameters	Results
Total number of compounds selected for screening	200
Number of compounds successfully soaked into individual crystals for data collection ^1^	154
Number of datasets that diffracted to better than 3 Å	88
Median resolution of collected datasets	2.2 Å
Number of hits ^2^	4

^1^ Excluding compounds that were poorly soluble in the crystallization conditions, and those leading to visible deterioration. ^2^ Well-ordered fragment binders, excluding those on crystal contacts.

**Table 2:** Crystallographic data collection and refinement statistics.

Binding Site	A (NS3-NS5 interface)		B (Interdomain hinge region)	
Fragment Hits	*1*	*2*	*3*	*4*
*Chemical name*	2,6-di-aminopurine	5-methylisatin	3-amino-1-[2-(4-chlorophenyl)ethyl] thiourea	2,4-dicholorobenzamide
*Structural formula*	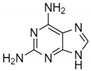	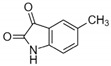	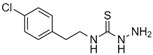	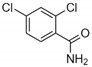
*Molecular weight (Da)*	150	160	230	190
**Data collection ^1^**				
*Cell parameters (space group P2_1_):*				
*a, b, c (Å)*	49.92, 73.22, 58.45	50.12, 73.35, 59.06	49.89, 73.46, 60.72	49.95, 74.17, 58.58
*β (°)*	90.85	91.66	99.51	90.7
*Resolution range (Å)*	46–1.9	46.6–2.2	49.21–2.35	49.8–1.80
*Unique reflections*	33305 (2063)	21559 (1783)	17920 (1742)	39559 (2239)
*Rmerge*	0.10 (1.21)	0.187 (1.63)	0.178 (1.62)	0.12 (1.74)
*Rmeas*	0.12(1.44)	0.22 (1.93)	0.21 (1.95)	0.14 (2.05)
*I/σ(I)*	10.3 (1.3)	6.8 (1.1)	7.4 (1.1)	11.1 (1.2)
*CC_1/2_*	0.99 (0.99)	0.97 (0.99)	0.99 (0.36)	0.99 (0.51)
*Completeness (%)*	99.6 (94.4)	98.7 (95.1)	98.6 (96.8)	99.6 (95.2)
*Redundancy*	6.8 (6.5)	6.9 (6.9)	7.0(6.5)	6.8 (6.6)
**Refinement**				
*Rwork*	0.194	0.219	0.202	0.185
*Rfree*	0.224	0.242	0.264	0.217
*No. of atoms:*				
*Protein*	3544	3544	3544	3434
*Ligand/ions*	81	69	107	43
*Water molecules*	354	276	149	491
*Mean B factor (Å^2^)*	36.6	44.5	52.0	28.1
*R.m.s. deviations:*				
*Bond lengths (Å)*	0.008	0.008	0.011	0.008
*Bond angles (°)*	0.96	0.96	0.91	0.94

^1^ Data in brackets are for the highest resolution (outer) shell.

## Appendix A

**Table A1:** Residues forming the binding sites A and B in flaviviral NS3 helicases.

Virus	ZIKV	DENV	Kunjin	JEV	YFV	MVEV	TBEV
PDB Code	This Work	2JLQ	2QEQ	2Z83	1YKS	2V8O	-
**Site A**	**R439**	**R**	**R**	**R**	K	**R**	**R**
	**I441**	**I**	**I**	**I**	A	**I**	E
	**N568**	**N**	T	T	E	S	A
	**N569**	**N**	**N**	**N**	H	**N**	**N**
	**T570**	Q	**T**	A	E	I	A
	**I571**	**I**	**I**	**I**	**I**	**I**	V
	**M572**	L	L	L	L	L	D
	**M595**	L	I	L	C	L	K
	**A597**	**A**	**A**	**A**	E	**A**	**A**
	**C600**	Y	Y	Y	S	Y	F
	**S601**	A	**S**	A	**S**	**S**	K
**Site B**	**V191**	**V**	**V**	**V**	**V**	**V**	**V**
	**A287**	**A**	**A**	**A**	**A**	**A**	**A**
	**H288**	**H**	**H**	**H**	**H**	**H**	**H**
	**F314**	**F**	**F**	**F**	A	**F**	L
	**T316**	**T**	**T**	**T**	**T**	**T**	**T**
	**P320**	**P**	**P**	**P**	**P**	**P**	**P**
	**E413**	**E**	I	S	I	**E**	**E**
	**T449**	**T**	**T**	**T**	S	**T**	**T**
	**S452**	**S**	**S**	**S**	**S**	**S**	**S**
	**Y508**	**Y**	**Y**	**Y**	**Y**	**Y**	**Y**
	**P510**	**P**	**P**	**P**	V	**P**	**P**

Comparison of the binding sites A (cyan background) and B (lime green background) was based on 3D structural superposition. For TBEV, no NS3-Hel structure is available and, therefore, the analysis was based on sequence alignment. Residues that are identical to those in ZIKV NS3-Hel are shown in bold.
